# Epidemic Genotype of *Coxiella burnetii* among Goats, Sheep, and Humans in the Netherlands

**DOI:** 10.3201/eid1805.111907

**Published:** 2012-05

**Authors:** Jeroen J.H.C. Tilburg, Hendrik-Jan I.J. Roest, Sylvain Buffet, Marrigje H. Nabuurs-Franssen, Alphons M. Horrevorts, Didier Raoult, Corné H.W. Klaassen

**Affiliations:** Canisius Wilhelmina Hospital, Nijmegen, the Netherlands (J.J.H.C. Tilburg, M.H. Nabuurs-Franssen, A.M. Horrevorts, C.H.W. Klaassen);; Central Veterinary Institute part of Wageningen UR, Lelystad, the Netherlands (H.I.J. Roest);; Université de la Méditerranée, Marseille, France (S. Buffet, D. Raoult)

**Keywords:** the Netherlands, Q fever, outbreak, *Coxiella burnetii*, MST, MLVA, bacteria, ruminants. goats, sheep, humans, multilocus variable-number tandem repeats analysis, multispacer sequence typing, genotypes, epidemic

**To the Editor:** The 2007–2010 Q fever epidemic among humans in the Netherlands was among the largest reported in magnitude and duration (*1*). The increase in human Q fever cases coincided with an increase in spontaneous abortions among dairy goats in the southeastern part of the Netherlands, an area that is densely populated with goat farms (*1*). Genotypic analyses of the involved isolates could confirm the possible link between the human and animal Q fever cases.

In previous studies, genotypic investigations of human and animal samples in the Netherlands were performed by using a 3-locus multilocus variable-number tandem repeats analysis (MLVA) panel and single-nucleotide polymorphism genotyping, respectively (*2**,**3*). The first study, performed on relatively few samples from a minor part of the affected area, showed that farm animals and humans in the Netherlands were infected by different but apparently closely related genotypes. More recently, genotyping by using a 10-locus MLVA panel provided additional information about the genotypic diversity of *Coxiella burnetii* among ruminants in the Netherlands: 1 dominant MLVA genotype was identified among goats and sheep throughout the entire affected Q fever area (*4*). A different panel of MLVA markers was applied to human samples (*5*). Four markers that are shared by both panels showed identical alleles in human and animal samples, again implicating goats and sheep as possible sources of the outbreak.

MLVA, which is based on relatively unstable repetitive DNA elements, is sometimes criticized for producing results that are too discriminatory or difficult to reproduce in different settings (*6*). Because of their instability, use of tandem repeats as genotyping targets can lead to problems with data interpretation and to overestimation of genotypic diversity by showing small variations in MLVA genotypes in isolates of otherwise identical background.

We used a more stable, sequence-based typing method, multispacer sequence typing (MST), on samples from humans and a group of ruminant animals (goats, sheep, and cattle) to establish a firmer correlation between Q fever cases in humans and animals (*7*). We identified MST genotypes using a Web-based MST database (http://ifr48.timone.univ-mrs.fr/MST_Coxiella/mst) containing genotypes from several countries in Europe. Ultimately, this study could answer the question of whether the current outbreak situation could have been caused by a specific *C. burnetii* strain in the ruminant population in the Netherlands.

Real-time PCR-positive specimens from 10 humans and 9 Q fever–positive specimens from goats and sheep collected from various locations throughout the affected area were used (*8*). We also included Q fever-positive specimens from cattle to rule out cattle as a possible source of Q fever infection. Five samples of cow’s milk and 1 bovine vaginal swab sample were analyzed (Table A1). MST33 was identified in 9 of 10 tested human samples and in the remaining 8 of 9 clinical samples from goats and sheep (Table A1). MST33 has been isolated incidentally in nonoutbreak situations in human clinical samples obtained in France during 1996, 1998, and 1999 and from a placenta of an asymptomatic ewe in Germany during 1992. All samples from cattle in the Netherlands, 1 goat, and cow’s milk contained genotype MST20. Genotype MST20 has also been identified in human clinical samples from France, in a cow’s placenta from Germany isolated in 1992 and in rodents from the United States isolated in 1958. In 1 human bronchoalveolar lavage sample, a novel (partial) MST genotype was found. This may be an incidental Q fever case unrelated to the outbreak situation. Because no historical genotyping data for the period before the outbreak of Q fever in the Netherlands are available, this explanation needs further research.

MST genotyping shows the presence of genotype MST33 in clinical samples from humans, goats and sheep. These results confirm that goats and sheep are the source of human Q fever in the Netherlands. Few worldwide genotyping studies have been conducted, and therefore information about a possible global persistence of this genotype is lacking. This study also indicates that the outbreak among humans is not linked to *C. burnetii* in cattle, although the infection is widespread among dairy herds in the Netherlands (*10*), exemplifying that most outbreaks are related to goats and sheep rather than to cattle. In conclusion, the increase in the number of Q fever cases in the Netherlands among humans most likely results from MST33 in the goat population in the Netherlands and could have been facilitated by intensive goat farming in the affected area and its proximity to the human population.

## Appendix

**Table A1:** *Coxiella burnetii* MST genotypes from humans and ruminants sampled during the Q fever outbreak, the Netherlands, 2008–2010*

Sample no.	Host	Source	Location	Year	C_t_ value	MST genotype†	Cox2	Cox5	Cox18	Cox20	Cox22	Cox37	Cox51	Cox56	Cox57‡	Cox61
Q001	Sheep	Vaginal swab	1	2008	25.7	33	7	5	1	–§	5	9	9	4	3	2
Q002	Sheep	Vaginal swab	1	2008	16.3	33	7	5	1	6	5	9	9	4	3	2
Q003	Sheep	Vaginal swab	1	2008	18.8	33	7	5	1	6	5	9	9	4	3	2
Q004	Lamb	Throat swab	1	2008	27.9	33	7	5	1	6	5	9	9	4	3	2
Q005	Lamb	Throat swab	1	2008	29.9	33	7	5	1	–	5	9	9	4	3	2
Q006	Lamb	Throat swab	1	2008	28.9	33	7	5	1	–	5	9	9	4	3	2
Q050	Human	BAL	2	2009	22.4	33	7	5	1	–	5	9	9	4	3	2
Q052	Human	Sputum	3	2009	20.7	33	7	5	1	–	5	9	9	4	3	2
Q054	Human	Sputum	3	2009	19.4	33	7	5	1	–	5	9	9	4	3	2
Q057	Human	Sputum	3	2009	20.6	33	7	5	1	–	5	9	9	4	3	2
Q063	Human	Sputum	4	2009	29.6	33	7	5	1	–	5	9	9	4	–	2
Q066	Human	Sputum	5	2009	27.7	33	7	5	1	–	5	9	9	4	–	2
Q076	Human	Aorta valve	6	2009	17.0	33	7	5	1	–	5	9	9	4	3	2
Q084	Human	Aorta valve	7	2008	17.0	33	7	5	1	–	5	9	9	4	3	2
Q107	Human	Aorta valve	5	2010	9.0	33	7	5	1	6	5	9	9	4	3	2
Q085	Goat	Placenta	8	2009	18.0	33	7	5	1	–	5	9	9	4	3	2
Q087	Goat	Placenta	9	2009	18.1	33	7	5	1	–	5	9	9	4	3	2
Q086	Goat	Placenta	9	2009	18.0	20	3	2	6	–	5	4	4	10	6	5
Q097	Cattle	Swab	10	2009	19.0	20	3	2	6	–	5	4	4	10	6	5
Q090	Cattle	Milk	11	2010	32.0	20	3	2	6	–	5	4	4	10	–	5
Q091	Cattle	Milk	12	2010	32.6	20	3	2	6	–	5	4	4	10	–	5
Q093	Cattle	Milk	13	2010	31.7	20	3	2	6	–	5	4	4	10	–	5
Q096	Cattle	Milk	14	2010	33.4	20	3	2	6	–	5	4	4	10	–	5
Q123	Cattle	Milk	15	2010	31.6	20	3	2	6	–	5	4	4	10	–	5
Q056	Human	BAL	16	2010	28.2	New	3	3	2	–	–	9	–	–	–	–
Dugway	NA	CP000733#	NA	NA	NA	20	3	2	6	1	5	4	4	10	6	5
RSA331	NA	CP000890#	NA	NA	NA	18	3	8	1	6	3	4	7	9	6	3
RSA493	NA	AE016828#	NA	NA	NA	16	3	8	5	3	4	1	6	7	6	5
CbuG Q212	NA	CP001019#	NA	NA	NA	21	2	1	4	6	2	3	1	11	1	1
CbuK Q154	NA	CP001020#	NA	NA	NA	8	5	4	2	5	1	5	3	3	4	4

*MST, multispacer sequence typing; C_t_, cycle threshold; BAL, bronchoalveolar lavage; NA, not applicable. †MST genotypes were identified by using the MST database (http://ifr48.timone.univ-mrs.fr/MST_Coxiella/mst). ‡Result obtained by using improved amplification primers for Cox57 (*9*). § –, no result was obtained. The lack of results may be explained by the significantly larger PCR product that is targeted, low quantity of DNA or to overall poor performance of the PCR amplification. ¶This combination of 4 alleles has not been observed and justifies the assignment of a new MST genotype. #GenBank accession number.
